# Roles of the peroxisome proliferator-activated receptors (PPARs) in the pathogenesis of diabetic kidney disease (DKD)

**DOI:** 10.1038/s41420-026-03117-8

**Published:** 2026-04-11

**Authors:** Zhi Zheng, Yunkuo Li, Yujie Pan

**Affiliations:** 1 Department of Orthopedics, Chengdu Integrated TCM & Western Medicine Hospital, Chengdu, People’s Republic of China; 2 Department of Targeting Therapy & Immunology and Laboratory of Animal Tumor Models, Cancer Center and State Key Laboratory of Respiratory Health and Multimorbidity and Frontiers Science Center for Disease-Related Molecular Network, West China Hospital, Sichuan University, Chengdu, China; 3 Department of Endocrinology and Metabolism, The First Hospital of Jilin University, Changchun, China

**Keywords:** Mechanisms of disease, Endocrine system and metabolic diseases

## Abstract

Diabetic kidney disease (DKD), a prevalent microvascular complication of diabetes, is a leading cause of chronic kidney disease and end-stage renal disease. Moreover, it plays a crucial role in the morbidity and mortality of diabetic patients. Peroxisome proliferator-activated receptors (PPARs), members of the nuclear hormone receptor superfamily, are key regulators of energy homeostasis, with three distinct subtypes identified: PPARα, PPARγ, and PPARβ/δ. They regulate gene transcription associated with energy metabolism, differentiation, inflammation, and cellular development. In recent years, the increasing incidence of DKD has intensified interest in elucidating the mechanisms and roles of PPARs in DKD. Unlike previous reviews—which focused primarily on individual PPAR subtypes or isolated pathological processes—this review adopts a unique “cell-type-specific perspective.” It systematically elucidates the distinct roles of all three PPAR isotypes (α, γ, and β/δ) across key renal cell types (podocytes, mesangial cells, tubular cells) in DKD. We also explore the potential influence of PPAR genetic polymorphisms on DKD susceptibility. Beyond conventional review frameworks, we propose an innovative therapeutic strategy encompassing PPAR-based multi-target synergistic approaches and nanotechnology-driven cell-specific targeted therapy. This offers novel directions to overcome current therapeutic bottlenecks.

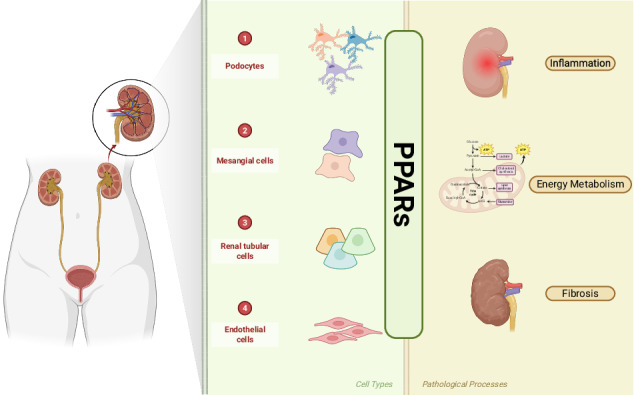

## Facts

- PPARs as Key Regulators of Energy Metabolism and Inflammation: PPARα/γ/δ subtypes orchestrate glycolipid metabolism, inflammatory responses, and cellular differentiation via transcriptional regulation, and their dysfunction is directly implicated in the progression of diabetic kidney disease (DKD).
- PPARγ agonists exert renal protective effects: Clinical evidence (e.g., thiazolidinediones) demonstrates that PPARγ activation reduces proteinuria and improves glomerular filtration barrier function in patients with early-stage diabetic kidney disease (DKD).
- Genetic polymorphisms influence susceptibility to diabetic kidney disease (DKD): Specific variants in PPAR genes (e.g., *PPARG* Pro12Ala) are significantly associated with the risk of renal complications in diabetic patients.

## Open questions

- In the self-reinforcing “metabolism-inflammation-fibrosis” cycle dominated by PPARs, how to design a multi-target synergistic strategy to break the limitations of single pathways? Is its efficacy superior to that of traditional single-drug therapy?
- How to precisely balance the bidirectional effects of PPAR subtypes in specific cell types?

## Introduction

Diabetes Mellitus (DM) is a complex metabolic syndrome defined by chronic hyperglycemia due to defects in insulin action (insulin resistance), insulin secretion, or both. This pathophysiological imbalance disrupts glucose homeostasis, resulting in chronic hyperglycemia. Elevated blood glucose levels in diabetic patients disturb both hemodynamic and metabolic homeostasis. As a result of this disturbed homeostasis, microvascular complications such as diabetic nephropathy (DN) occur [1]. DN, also known as diabetic kidney disease (DKD), is a leading complication associated with diabetes. More than 25% to 40% of patients with type 1 or type 2 diabetes (T2D) develop nephropathy after 20 to 35 years [2]. The clinical hallmarks of DKD include persistent hyperglycemia (even in the prediabetic phase), a progressively decreased glomerular filtration rate, increased urinary albumin excretion (≥300 mg/d), and a progressive decline in renal function, ultimately leading to end-stage kidney failure [3]. Pathologically, DKD progresses through multiple stages: an initial hyperglycemic environment induces renal hemodynamic changes and metabolic disorders, leading to glomerular hyperfiltration and proteinuria; persistent metabolic stress and accumulation of advanced glycation end products (AGEs) exacerbate mitochondrial dysfunction and oxidative stress, triggering podocyte injury, mesangial matrix expansion, and tubulointerstitial inflammation; eventually, profibrotic factors such as transforming growth factor-β (TGF-β) drive the excessive extracellular matrix (ECM) deposition, resulting in progressive glomerulosclerosis and interstitial fibrosis, ultimately causing irreversible renal function loss [4–7]. Risk factors for developing DKD include smoking, obesity, non-Caucasian ethnicity, systemic or glomerular hypertension, older age, and poor control of glycemia and lipid levels [8]. Interstitial inflammation and fibrosis are hallmark features of DKD, which is widely recognized as a major contributor to the development of end-stage renal disease (ESRD) and a significant risk factor for cardiovascular diseases [9]. DKD affects at least 30% of patients with diabetes and imposes a significant burden on public health [10]. A separate study revealed that 60.3% of patients with DKD and stage 4 chronic kidney disease rapidly progressed to ESRD or death, of whom 10.9% died [11]. This suggests that despite improving treatments, DKD continues to pose significant challenges to patient health and healthcare systems. Many patients still face the threat of ESRD, highlighting the limitations of current treatment strategies. Therefore, developing more effective therapeutic targets and strategies to slow the progression of DKD and improve patients’ quality of life has become a crucial direction in medical research today, as well as a key challenge that needs to be addressed.

Peroxisome proliferator-activated receptors (PPARs), belonging to the nuclear hormone receptor family, comprise three subtypes: α, β/δ, and γ. Each subtype exhibits unique metabolic regulatory activities, ligand specificity, and tissue distribution, and is encoded by an individual gene [12]. PPARα (NR1C1) is expressed in the kidney, heart, brown adipose tissue, liver, and skeletal muscle. It uses polyunsaturated fatty acids and arachidonic acid metabolites as natural ligands, while fibrates serve as synthetic ligands. Existing studies have demonstrated that PPARα agonists exhibit significant therapeutic effects on DKD, primarily by reducing albuminuria and inhibiting renal fibrosis [13]. PPARβ/δ (NR1C2) is expressed in skin, brain and adipose tissue and is associated with muscle development and oxidative capacity [14]. PPARγ (NR1C3) is widely expressed and plays a crucial role in adipogenesis [15]. While fatty acids and eicosanoids can serve as ligands for PPARγ, the main ligands with physiological relevance for this receptor remain unclear [16]. The physiological and medical significance of PPARγ was emphasized by the discovery that thiazolidinediones (TZDs) drugs, renowned for improving insulin sensitivity, serve as high-affinity ligands for PPARγ [17–20].

Understanding the function of PPARs can be facilitated by comparing their mechanism of action to that of other members within the superfamily. The glucocorticoid receptor, along with other steroid hormone receptors, is primarily localized in the cytoplasm, where it forms complexes with cytoplasmic chaperones. Ligand binding induces homodimerization, followed by nuclear translocation, enabling interaction with DNA as homodimers and the subsequent recruitment of co-activator complexes to activate transcription of target genes. In contrast, PPARγ exhibits distinct behavior: it constitutively localized in the nucleus and forms a stable heterodimer with the retinoid X receptor (RXR), which is pre-bound to DNA even in the absence of ligand. In the absence of ligand, the PPARγ/RXR heterodimer recruits co-repressors and histone deacetylases, thereby repressing target gene expression. Upon ligand binding, co-repressors are released, and co-activators, along with histone acetyltransferases, are recruited, resulting in a co-activator complex that enhances transcription. Genome-wide chromatin immunoprecipitation analyses have shown that PPARγ/RXR is capable of binding to more than 5000 sites in the genome [21]. Nonetheless, transcriptome analysis demonstrated that the quantity of activated genes is significantly lower [22]. Consequently, while the range of potential target genes is extensive, the actual changes in gene expression are more limited and specific to certain cell types.

**Fig. 1:**
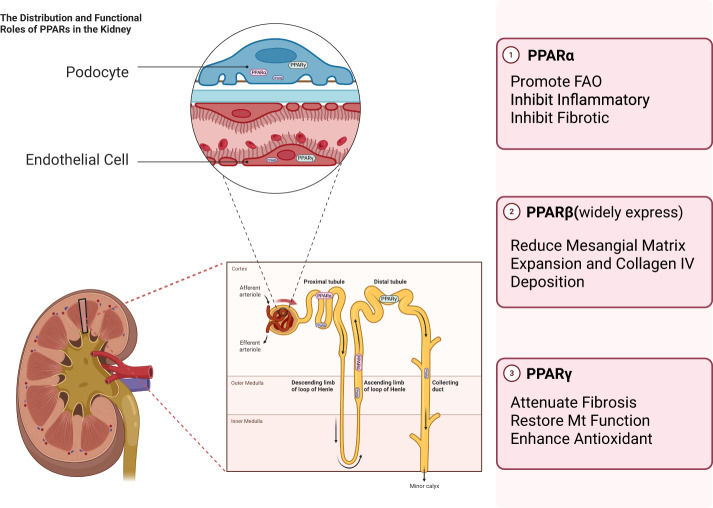
The three PPAR subtypes exhibit a compartment-specific distribution in the kidney and function synergistically to exert protective effects. PPARα, enriched in proximal tubules, promotes FAO and suppresses inflammation and fibrosis. PPARγ, located in collecting ducts and glomerular cells, attenuates fibrosis, restores mitochondrial function, and enhances antioxidant defense. PPARβ/δ, widely expressed throughout the kidney, confers protection by reducing albuminuria and inflammation. PPAR peroxisome proliferator-activated receptor, PPARγ peroxisome proliferator-activated receptor gamma, PPARα peroxisome proliferator-activated receptor α, PPARβ peroxisome proliferator-activated receptor β, FAO Fatty acid oxidation; (Created by Biorender.com).

Given the central role of PPARs in regulating glucose and lipid metabolism, inflammatory responses, and oxidative stress—processes that are key drivers of DKD development—the role of PPARs in this disease is increasingly recognized. Studies have confirmed that all PPAR subtypes exhibit differential expression in the kidney [23–25] (Fig. 1). PPARα is predominantly expressed in the proximal tubules and the thick ascending limb of the medulla, while PPARγ is expressed in glomerular mesangial cells, pelvic urothelium, and medullary collecting ducts. PPARβ exhibits ubiquitous low-level expression across all nephron segments. This cell-specific distribution allows PPARs to directly regulate local metabolic, inflammatory, and fibrotic processes within the kidney, thereby playing a crucial modulatory role in the pathophysiology of DKD. In glomerular mesangial cells, PPARα ameliorates oxidative stress and inhibits glomerular and tubulointerstitial fibrosis, PPARβ/δ promotes fatty acid oxidation (FAO), and PPARγ exerts anti-apoptotic and antioxidant effects. In proximal tubules, PPARα increases FAO and reduces inflammation, PPARβ exhibits anti-inflammatory activity, and PPARγ reduces insulin resistance and exerts anti-inflammatory effects. In podocytes, PPARα attenuates albuminuria and improves insulin resistance, while PPARγ activates autophagy and inhibits tubulointerstitial fibrosis. This matching of cell-specific distribution and functions enables the PPAR family to synergistically address metabolic disorders, inflammation, and fibrosis during DKD progression at the multicellular level [26]. PPARα activation promotes FAO-related gene expression to reduce renal lipid accumulation while simultaneously suppressing inflammatory responses via inhibition of nuclear factor-kappa B (NF-κB) nuclear translocation. This dual mechanism of action establishes PPARα as a critical therapeutic target for addressing both lipotoxic and inflammatory pathways in DKD [27]. Activation of PPARγ is recognized as a protective mechanism against mitochondrial dysfunction in podocytes, thereby mitigating the progression of DKD [28]. Additionally, PPARγ modulates the expression of fibrotic factors, including connective tissue growth factor (CTGF), TGF-β, and collagen type IV alpha 4. It also regulates the activity of various metalloproteinases, collectively contributing to the attenuation of glomerular and tubulointerstitial fibrosis in DKD [29]. In contrast, the precise role of PPARβ/δ in DKD and its potential therapeutic value remain relatively underexplored, with limited research evidence available to date.

Despite existing reviews on PPARs in DKD, most focus on a single isoform (e.g., PPARγ) or an isolated pathological process, lacking a systematic analysis of the entire PPAR family. Furthermore, the cell-specific mechanisms linking distinct PPAR isoforms to characteristic DKD pathologies remain fragmented. In light of these gaps, this review makes the following novel contributions: (1) an integrative framework that connects PPAR subtypes (α, γ, β/δ), renal parenchymal cells (proximal tubules, mesangial cells, podocytes), and DKD pathological processes (lipotoxicity, inflammation, fibrosis). This framework is structured around the core logic of cell-type-specific expression, followed by subtype-specific functional regulation, and ultimately targeting key pathological processes in DKD. Specifically, PPARα (predominantly expressed in proximal tubules) exerts regulatory effects on lipotoxicity and inflammation; PPARγ (localized in mesangial cells) modulates fibrosis and mitochondrial dysfunction; and PPARβ/δ (ubiquitously expressed at low levels across nephron segments) complements the functional roles of the α/γ subtypes in basal metabolic regulation. By systematically synthesizing the hierarchical and complementary regulatory relationships between the three PPAR subtypes and their corresponding renal cellular targets during DKD progression, this framework addresses the fragmented understanding in previous reviews that focused on single isoforms or isolated pathological mechanisms. (2) innovative therapeutic strategies built upon the cooperative mechanisms of the three subtypes, including cell-type-specific targeting and multi-target synergistic therapy. These approaches overcome the limitations of conventional single-target therapies and provide a theoretically sound foundation for precision treatment of DKD.

## Cell-specific mechanisms of PPARs in DKD

### Podocytes

Podocytes, terminally differentiated epithelial cells of the glomerular epithelium, are highly susceptible to injury under pathological stimuli such as high glucose (HG). This injury is characterized by the flattening, widening, and retraction of foot processes, compromising the integrity of the glomerular filtration barrier and ultimately contributing to the development of DKD [30]. Recent studies have demonstrated that the PPARs signaling pathway plays a key role in podocyte injury and apoptosis in DKD, and its regulatory mechanisms have been widely recognized.

#### PPARγ protective mechanisms

Podocytes, as specialized glomerular cells, exhibit higher basal autophagic activity than other renal cell types [31]. Impaired podocyte autophagy has been observed in both DKD mice and patients [32, 33]. A study by Xie et al. (2024) [34] demonstrated that the expression of nuclear receptor coactivator 3 (NCOA3) was reduced in the kidneys of DKD mice and patients. Since PPARγ, a ligand-activated transcription factor, relies heavily on coactivators for transcriptional activity, the same group generated a podocyte-specific NCOA3 knockout mouse model. NCOA3 knockout exacerbated podocyte injury, increased urinary albumin excretion, and worsened glomerulosclerosis, while also decreasing autophagy in the DKD model [34]. Conversely, overexpression of NCOA3 alleviated renal injury, suggesting its protective role against podocyte injury. Mechanistically, NCOA3 promotes the binding of PPARγ to the Fyn promoter, thereby suppressing Fyn transcription in a PPARγ-dependent manner and restoring podocyte autophagy [34]. PPARγ also exerts protective effects through antioxidant and anti-apoptotic pathways. Klotho acts as a downstream effector of PPARγ, which promotes Klotho expression in podocytes by binding to its gene promoter [35]. In HG-cultured podocytes, PPARγ activation induces Klotho up-regulation, which in turn promotes Forkhead transcription factor O1 (FoxO1) dephosphorylation and nuclear translocation. This cascade activates the antioxidant enzyme superoxide dismutase 2 (SOD2), ultimately leading to reactive oxygen species (ROS) scavenging and the maintenance of redox homeostasis [36]. Thus, the PPARγ-Klotho-FoxO1 pathway effectively suppresses HG-induced podocyte apoptosis by alleviating oxidative stress, thereby preventing the onset and progression of DKD [36]. In the same HG model, Perilipin2 attenuates podocyte apoptosis by upregulating podocyte-associated proteins and downregulating apoptosis-related proteins through PPARγ signaling pathway activation, thereby serving as a podocyte protector in DKD [37]. Furthermore, pigment epithelium-derived factor (PEDF) blocks AGE-induced apoptosis in mouse podocytes by inhibiting receptor for advanced glycation end product **(**RAGE) expression and subsequent ROS generation, partly through PPARγ activation [38]. Moreover, PPARγ activation exerts a protective effect by preventing podocyte mitochondrial dysfunction and impeding the progression of DKD [28]. These findings collectively indicate that PPARγ activation protects podocytes through multidimensional mechanisms, including the inhibition of mitochondrial dysfunction, promotion of autophagy, and regulation of redox homeostasis. These actions establish PPARγ as a key therapeutic target for alleviating podocyte injury and slowing DKD progression.

#### The detrimental role of PPARγ

Although PPARγ generally exhibits well-established protective effects in DKD, it can also mediate detrimental effects under certain conditions. Zhou et al. (2017) [39] demonstrated that the miR-27a/PPARγ/β-catenin axis plays a critical role in driving podocyte injury and renal dysfunction in DKD. Mechanistically, miR-27a exerts dual regulatory modes on PPARγ: on one hand, it directly targets the 3′-untranslated region (3′-UTR) of *PPARγ* mRNA to repress its transcriptional expression; on the other hand, it indirectly stimulates PPARγ phosphorylation, thereby activating the β-catenin signaling pathway [39]. This coordinated regulation triggers β-catenin-dependent cellular reprogramming in podocytes, including loss of podocyte-specific markers, promotion of epithelial-mesenchymal transition (EMT), and increased apoptosis. Zhou et al. (2017) [39] further validated this mechanism in vivo, showing that miR-27a overexpression reduces podocyte number and disrupts podocyte architectural integrity in diabetic rats. These changes ultimately lead to impaired renal function, as evidenced by increased urinary albumin excretion and decreased creatinine clearance rate. Crucially, this molecular mechanism was verified in renal biopsies from DKD patients: the upregulation of miR-27a and the activation of the PPARγ/β-catenin signaling pathway in podocytes isolated from these biopsies [39]. In conclusion, miR-27a fosters the progression of DKD by inducing podocyte injury via PPARγ-mediated β-catenin activation [39]. This signaling axis provides new insights into the core mechanisms of podocyte injury in DKD and the development of innovative combinational therapeutic strategies.

#### PPARα: metabolic regulation

PPARα also plays a crucial role in protecting podocytes in DKD. First, PPARα activation orchestrates podocyte homeostasis through a dual metabolic-antioxidant mechanism. Kim et al. (2018) [40] focused on the expression of adiponectin receptors (AdipoR1/2) and associated intracellular signaling pathways in 27 type 2 diabetic patients. It also explored pharmacological adiponectin receptors (AdipoRs) activation effects in three models: human glomerular endothelial cells (GECs), male C57BLKS/J db/db mice, and murine podocytes [40]. In HG-treated human GECs and murine podocytes, AdipoR1/2 activation significantly increased intracellular Ca²⁺ levels, activating the Ca²⁺/LKB1-AMPK/PPARα pathway. This reduced lipotoxicity, oxidative stress, and apoptosis, improved endothelial dysfunction and podocyte injury, thus slowing DKD progression. It is worth emphasizing that the Ca²⁺/LKB1-AMPK/PPARα signaling axis, activated by pharmacological AdipoRs stimulation, not only halts DKD progression in the kidney but also confers cardioprotective effects via conserved pathways. Additionally, pharmacological activation of AdipoRs activates AMPK and PPARα, thereby exerting antidiabetic effects through their downstream effectors—ACC, SREBP-1c, and PGC-1α [40].

Similarly, PPARα protects DKD by promoting FAO in podocytes. In adriamycin-induced DKD models, the E3 ubiquitin ligase tripartite motif containing 63 (Trim63) mediates the ubiquitination and proteasomal degradation of PPARα, leading to FAO deficiency and mitochondrial dysfunction. This sequence of events results in podocyte injury, thereby exacerbating proteinuria and glomerular damage associated with DKD [41]. On the other hand, rho-associated coiled-coil containing protein kinase 2 (ROCK2), functioning as an endogenous suppressor of PPARα, rewires cellular programs to exert negative control over the transcription of FAO-related genes, ultimately inducing podocyte apoptosis. Podocyte-specific *ROCK2* knockout studies showed that *ROCK2* deletion restores PPARα-mediated expression of FAO genes, improves fatty acid metabolism, and alleviates diabetic kidney injury, indicating that ROCK2 acts as a negative regulator of podocyte energy production and cellular homeostasis [42].

**Fig. 2:**
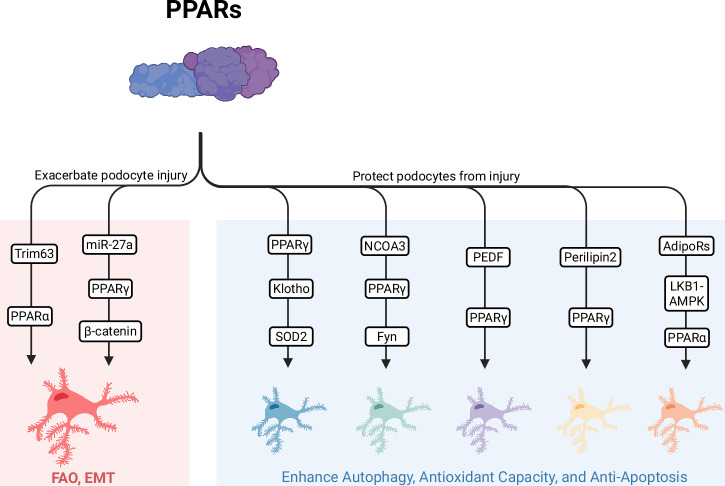
PPARs play a central role in regulating podocyte homeostasis. Protective Pathways (Right): NCOA3 suppresses the transcription of Fyn in a PPARγ-dependent manner, thereby promoting podocyte autophagy. PPARγ enhances antioxidant capacity through the Klotho-Forkhead Transcription Factor O1 (FoxO1) axis; meanwhile, PEDF and Perilipin 2 exert anti-apoptotic effects via PPARγ. As a key molecule downstream of AdipoRs, PPARα maintains metabolic homeostasis through the AMPK pathway. Damaging Pathways (Left): miR-27a activates β-catenin via PPARγ, inducing EMT and podocyte apoptosis. Additionally, Trim63 mediates the ubiquitination of PPARα, resulting in FAO deficiency and mitochondrial dysfunction. PPARs Peroxisome proliferator-activated receptors, PPARγ peroxisome proliferator-activated receptor gamma, PPARα peroxisome proliferator-activated receptor α, SOD2 superoxide dismutase 2, NCOA3 nuclear receptor coactivator 3, PEDF pigment epithelium-derived factor, LKB1 liver kinase B1, AMPK adenosine monophosphate-activated protein kinase, EMT epithelial-mesenchymal transition, FAO fatty acid oxidation, AdipoRs adiponectin receptors; (Created by Biorender.com).

These findings reveal that nuclear receptors PPARγ and PPARα exert protective functions in podocytes through distinct yet functionally synergistic pathways regulating autophagy, antioxidant responses, and energy metabolism. However, their activities are subjected to specific pathological antagonism—miR-27a suppresses PPARγ, while PPARα is inhibited via Trim63-mediated proteasomal degradation and ROCK2-mediated transcriptional repression (Fig. 2). These molecular events highlight the pivotal role of dysregulated nuclear receptor signaling in podocyte injury during DKD, providing an integrative, cross-molecular perspective for understanding the pathological mechanisms underlying glomerular filtration barrier disruption.

The PPARγ and PPARα signaling pathways in podocytes serve as the first line of defense against the core pathological processes of DKD. They maintain podocyte viability by regulating metabolism (PPARα-mediated FAO), oxidative stress (the PPARγ-mediated Klotho-FoxO1-SOD2 axis), and autophagy, thereby preserving the integrity of the glomerular filtration barrier and delaying the initiation of fibrosis. However, podocyte injury and detachment are merely the starting point of glomerular lesions. The loss of podocyte function directly alters intraglomerular hemodynamics and intercellular crosstalk, thereby activating and exacerbating pathological responses in mesangial cells located at the core of the glomerulus—particularly driving the core processes of inflammation and fibrosis.

### Mesangial cells

**Fig. 3:**
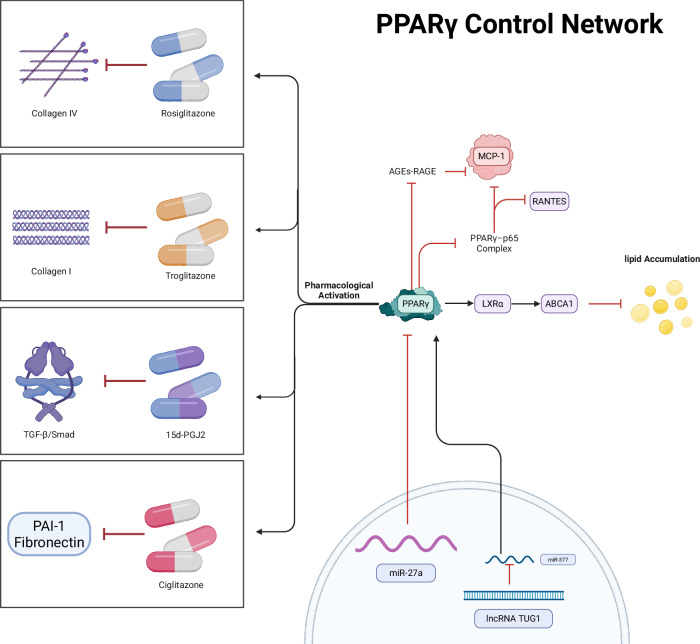
In DKD, PPARγ integrates key pathological signals in mesangial cells and directly regulates clinically relevant renal injury. PPARγ mediates anti-inflammatory effects by inhibiting the AGE-RAGE pathway and NF-κB pathway. Moreover, PPARγ exerts antifibrotic effects by antagonizing TGF-β signaling and downregulating Collagen I, Collagen IV, PAI-1, and fibronectin. Furthermore, PPARγ alleviates lipid accumulation through the LXRα-ABCA1 axis. The activity of PPARγ is controlled by epigenetic factors such as miR-27a and lncRNA TUG1. PPARγ agonists (e.g., rosiglitazone, troglitazone) orchestrate the aforementioned pathways to collectively exert renoprotective effects and delay the functional decline of DKD. PPARγ peroxisome proliferator-activated receptor gamma, DKD diabetic kidney disease, Collagen IV type IV collagen, Collagen I type I collagen, TGF-β Transforming growth factor-β, 15d-PGJ2 15-deoxy-Δ^12,14^-prostaglandin J2, PAI-1 plasminogen activator inhibitor-1, MCP-1 monocyte chemoattractant protein-1, RANTES Regulated upon activation, normal T-cell expressed and secreted, AGE advanced glycation end product, RAGE receptor for advanced glycation end product, LXRα liver X receptor α, ABCA1 ATP-binding cassette subfamily A member 1, lncRNA TUG1 long noncoding RNA taurine-upregulated gene 1; (created by Biorender.com).

In DKD, the inflammatory activation and fibrosis of glomerular mesangial cells are pivotal pathological events that drive glomerulosclerosis. In addition to their roles in podocytes, PPARs also act in mesangial cells to counteract the three principal pathogenic factors of DKD: chronic inflammation, metabolic dysregulation, and fibrotic remodeling (Fig. 3).

#### PPARγ and inflammation

Studies have shown that several drugs, particularly PPARγ agonists, exert protective effects in DKD, acting primarily through PPARγ as the critical effector hub [43–46]. PPARγ targets the AGE-RAGE signaling axis through dual pharmacological strategies. Angiotensin receptor blockers (e.g., telmisartan) and calcium channel blockers (e.g., nifedipine) activate PPARγ, which in turn suppresses *RAGE* gene expression [47, 48]. This effect reduces monocyte chemoattractant protein-1 (MCP-1)-mediated inflammatory cell chemotaxis and inhibits AGEs-induced mesangial cell injury, ultimately conferring renoprotective effects during DKD progression [47, 48]. The anti-AGE-RAGE pathway interacts with PPARγ‘s direct regulation of the classical inflammatory hub NF-κB, achieving precise synergy at the MCP-1 target. Mechanistically, rather than merely acting as a downstream target, PPARγ actively intercepts inflammatory signaling. For example, in a rat mesangial cell model, PPARγ agonists effectively inhibit inflammatory responses and improve DKD by preventing the interaction and complex formation between PPARγ and p65 [49]. This suppresses NF-κB dependent, tumor necrosis factor-alpha (TNF-α)-induced expression of RANTES (a chemokine expressed and secreted upon T-cell activation) and MCP-1 [49]. Together, these mechanisms establish a “bidirectional interception” of the inflammatory cascade within the glomerular microenvironment. PPARγ‘s anti-inflammatory effects also synergize with its anti-fibrotic functions. In streptozotocin (STZ)-induced diabetic rat models, rosiglitazone-mediated activation of PPARγ does not improve hyperglycemia or glomerular basement membrane thickening but significantly reduces albuminuria and tubulointerstitial fibrosis. This occurs through reduced type IV collagen (Collagen IV) expression, decreased renal macrophage infiltration, and inhibition of AGE-induced p38 mitogen-activated protein kinase (p38 MAPK) activation, nitrite release, and inducible nitric oxide synthase expression in mesangial cells [50–52], demonstrating glucose-independent renoprotection. Conversely, this protective signaling can be hijacked by the diabetic milieu. The abnormally activated cannabinoid receptor 1 (CB1R) signaling pathway in a hyperglycemic environment represents a critical antagonistic mechanism. In STZ-induced diabetic rats, hyperglycemia significantly upregulates CB1R expression in mesangial cells [53]. Activated CB1R suppresses PPARγ2 signaling in mesangial cells, thereby activating the inflammatory regulator SOCS3 and the fibrogenic transcription factor c-Jun. This increases synthesis of proinflammatory cytokines and ECM in mesangial cells, exacerbating cell dysfunction and accelerating the pathological progression of DKD [53].

#### PPARγ and fibrosis

Beyond its role in suppressing inflammation and the AGE-RAGE pathway, PPARγ also modulates the metabolism of ECM in mesangial cells by targeting the TGF-β signaling pathway. This achieves multidimensional intervention in DKD-associated fibrosis. As key mediators of pathological ECM deposition in DKD, mesangial cells, when driven by TGF-β signaling, overproduce collagen [54, 55]. PPARγ restores ECM homeostasis through multiple regulatory strategies, thereby mitigating glomerulosclerosis and renal dysfunction in DKD. Specifically, in mouse mesangial cells cultured under high glucose (25 mM), PPARγ activation by troglitazone effectively inhibits the TGF-β1-induced increase in type I collagen (Collagen I) mRNA and protein expression, thereby reducing ECM accumulation [56]. Furthermore, incubation of mesangial cells with synthetic or natural PPARγ agonists—including 15-deoxy-Δ^12,14^-prostaglandin J2 (15d-PGJ2), ciglitazone, or troglitazone—suppresses the TGF-β1-mediated expression of α-smooth muscle actin, plasminogen activator inhibitor-1 (PAI-1), and fibronectin [57]. Specifically, 15d-PGJ2 induces PPARγ binding to the peroxisome proliferator response element in the hepatocyte growth factor promoter in mesangial cells. PPARγ agonists also promote c-met receptor tyrosine phosphorylation, induce the expression of the Smad co-repressor TG-interacting factor, and block TGF-β/Smad-driven gene transcription in mesangial cells [57]. Moreover, in STZ-induced DKD rats, activation of PPARγ alleviates the inhibition of plasminogen and matrix metalloproteinases (MMPs) imposed by PAI-1, promoting ECM degradation. This mechanism concurrently suppresses mesangial cell hyperproliferation in early DKD, achieving a dual effect: inhibition of ECM synthesis and promotion of its degradation [51].

Epigenetic mechanisms further amplify PPARγ‘s antifibrotic effects. In STZ-induced diabetic rats, miR-27a negatively regulates PPARγ expression by binding to its 3’-UTR, exacerbating renal collagen deposition [58]. Conversely, in db/db DKD mice, long noncoding RNA taurine-upregulated gene 1 (lncRNA TUG1) functions as an endogenous sponge for miR-377. By downregulating miR-377, TUG1 relieves its repressive effect on the target gene *PPARG*, thereby attenuating ECM accumulation in mesangial cells [59].

Additionally, in cultured rabbit mesangial cells, TZDs-activated PPARγ upregulates liver X receptor α (LXRα) expression [23, 60–62]. LXRα-driven enhancement of adenosine triphosphate (ATP)-binding cassette transporter A1 expression promotes cholesterol removal from mesangial cells, improving intracellular lipid accumulation in DKD [62]. This demonstrates that PPARγ‘s structural protection against fibrosis is intricately linked to the restoration of cellular lipid metabolism.

#### Synergistic effects of other PPAR subtypes

In addition to the core anti-fibrotic pathway mediated by PPARγ, the protective mechanisms of mesangial cells also involve synergistic effects of other subtypes within the PPARs family and associated signaling pathways. Resveratrol, as a natural activator of the AMPK-SIRT1-PGC-1α signaling axis, intervenes in the progression of DKD via two pathways. One pathway activates the PPARα-ERR-1α-SREBP1 axis to promote renal lipid metabolism and clearance. The other targets FOXO3a-mediated oxidative stress and cell death, thereby alleviating HG-induced mesangial cell damage. These pathways may suppress lipotoxicity-induced apoptosis and oxidative stress in the kidney, preventing DKD progression in db/db mice [63]. The dual PPARα/γ agonist tesaglitazar further expands the anti-fibrotic strategy by suppressing HG-induced expression of type I and IV collagen genes. This inhibits abnormal ECM deposition, significantly improving renal glomerular fibrosis and albuminuria in db/db mice [64]. This antifibrotic mechanism functionally complements the PPARδ pathway. Under AGE stimulation, the PPARδ agonist enhances anti-inflammatory effects by upregulating glucagon-like peptide-1 receptor. It also inhibits AGE-RAGE axis activation, preserving mesangial cell viability [65].

The PPARs family in mesangial cells serves as key nodes for intervening in the metabolism-inflammation-fibrosis axis. PPARs exert anti-inflammatory effects primarily by inhibiting the NF-κB and AGE-RAGE axes, and direct anti-fibrotic effects through antagonizing the TGF-β signaling pathway and regulating ECM synthesis and degradation. These actions precisely target the core processes driving glomerulosclerosis. Therefore, targeting mesangial cell PPARs can effectively suppress intra-glomerular inflammation and matrix expansion. However, the pathological progression of DKD is not confined to the glomeruli. Persistent inflammatory and metabolic dysregulation signals spill over into the tubular region, initiating tubulointerstitial inflammation and fibrosis. This is a critical step determining DKD progression to renal failure.

### Renal tubular cells

**Fig. 4:**
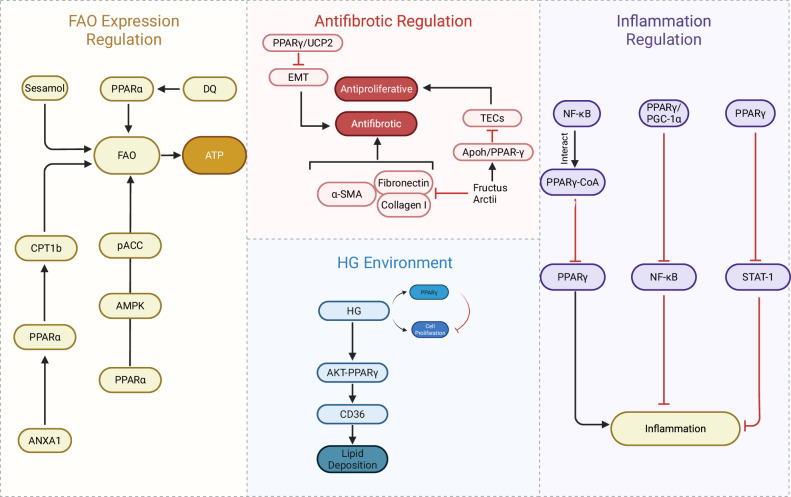
In the renal tubules of DKD, PPARs function as a signaling hub to orchestrate metabolic, fibrotic, and inflammatory responses. PPARα enhances FAO by activating AMPK and upregulating CPT1b, thereby alleviating lipotoxicity. On the other hand, PPARγ inhibits EMT and suppresses TECs by upregulating the Apoh/UCP2 axis; PPARγ also antagonizes NF-κB and STAT-1 to mitigate inflammation. Conversely, the HG environment upregulates the fatty acid translocase CD36 via the AKT-PPARγ axis, resulting in lipid deposition and inducing lipotoxic injury in renal tubules. PPARγ peroxisome proliferator-activated receptor gamma, PPARα peroxisome proliferator-activated receptor α, TECs tubular epithelial cells, UCP2 uncoupling protein 2, KLF6 Krüppel-like factor 6, ANXA1 Annexin A1, CPT1b carnitine palmitoyltransferase 1b, DQ dasatinib and quercetin, NF-κB nuclear factor-kappa B, EMT epithelial-mesenchymal transition, FAO fatty acid oxidation, ATP adenosine triphosphate, pACC phosphorylated Acetyl-CoA Carboxylase, AMPK adenosine monophosphate-activated protein kinase, α-SMA alpha-smooth muscle actin, Collagen I type I collagen, HG high glucose, STAT-1 signal transducer and activator of transcription 1, PGC-1α peroxisome proliferator-activated receptor gamma coactivator 1 alpha; (created by Biorender.com).

Tubular injury is another critical determinant of progressive renal injury in DKD [66]. Tubular epithelial cells (TECs), particularly proximal tubular epithelial cells (PTECs), serve as key regulatory mediators of renal inflammation and fibrosis. Under injury conditions, TECs release chemokines that recruit macrophages and monocytes to the site of injury. Their activation and aggregation intensify the local inflammatory cascade, ultimately leading to tissue damage and fibrosis [67, 68]. In this process, PPARs serve as critical regulatory nodes within TECs, playing a pivotal role in renal tubular pathophysiology in DKD (Fig. 4).

#### PPARγ: antifibrotic, anti-inflammatory, and lipid metabolic regulation

In db/db mice, Fructus arctii upregulates the expression of Apoh and PPARγ. It inhibits the abnormal proliferation of TECs by activating the Apoh/PPARγ signaling pathway. It also suppresses the expression of fibrosis markers, including Fibronectin, α-SMA, and Collagen I, thereby attenuating DKD progression. This effect depends on the mediation of Apoh, as silencing Apoh eliminates Fructus arctii’s antifibrotic and antiproliferative benefits [69]. In the db/db diabetic mouse, Uncoupling protein 2 (UCP2) and its upstream gene PPARγ were found to be significantly upregulated. The PPARγ/UCP2 pathway attenuates renal EMT and alleviates tubulointerstitial fibrosis [70]. Additionally, in vitro experiments showed that HG induces PPARγ upregulation in proximal tubular cells (e.g., HK-2 cells), forming a negative feedback loop to restrict excessive cell proliferation and attenuate the progression of DKD [71].

PPARγ’s anti-inflammatory mechanisms involve multi-pathway synergy. First, NF-κB interacts directly with PPARγ and its coactivators. This interaction downregulates PPARγ transcriptional coactivators, thereby exacerbating renal inflammatory injury and accelerating DKD progression. Conversely, PPARγ/PGC-1α activators effectively block the NF-κB axis in HK-2 cells and db/db DKD mice, significantly alleviating inflammation [72]. Second, AGE-driven upregulation of adhesion molecules and chemokines in renal tubular cells promotes the migration of inflammatory cells into the renal interstitium during diabetic tubulopathy. PPARγ ligands counteract this proinflammatory cascade by selectively inhibiting STAT-1 phosphorylation, a mechanism independent of classical NF-κB and MAPK pathways [73]. Furthermore, Qi et al. (2009) [74] revealed that in both in vivo (STZ-induced diabetic Sprague-Dawley rats) and in vitro (human kidney proximal tubular cells) models of DKD, the overexpression of Krüppel-like factor 6 (KLF6) increased thioredoxin-interacting protein expression and promoter activity. Co-treatment with PPARγ agonists suppresses these effects. As an endogenous regulator of PPARγ, KLF6 is upregulated in DKD and exacerbates renal injury by inhibiting PPARγ. However, in vitro studies show PPARγ agonists do not directly modulate KLF6 expression, suggesting that the two factors contribute to the progression of DKD through distinct pathways [74].

PPARγ can exert contradictory effects on lipid metabolism. HG activates the AKT-PPARγ pathway, resulting in CD36 upregulation and subsequent lipid deposition in HK-2 cells. Inhibiting this pathway alleviates lipotoxicity in diabetic kidneys [75].

#### PPARα: metabolic homeostasis and cytoprotection

PPARα has also attracted significant attention in DKD, as the kidney, being a metabolically active tissue, primarily relies on FAO for energy production. By enhancing FAO in renal tissues and cells, PPARα mitigates lipotoxicity [76]. Clinical studies provide direct evidence for this mechanism. Herman et al. found that *PPARA* and other genes involved in the FAO pathway were downregulated in kidney biopsies from DKD patients [77]. This clinical phenomenon has been thoroughly elucidated at the molecular mechanism level. Studies have demonstrated that Annexin A1 (ANXA1) deletion aggravates renal injuries in STZ-induced/high-fat diet diabetic mice, including albuminuria, tubulointerstitial lesions, and mesangial matrix expansion. In human TECs treated with HG and palmitic acid, ANXA1 silencing significantly reduced mitochondrial FAO activity by suppressing PPARα and its downstream target carnitine palmitoyltransferase 1b (CPT1b). This led to intracellular lipid accumulation and injury in HK-2 cells, thereby exacerbating lipotoxicity-mediated tubular damage in DKD [78].

Given the role of PPARα inactivation in tubular injury, its activation has therapeutic potential. Study findings demonstrated that PEDF activates the ATGL-PPARα pathway, which promotes fatty acid β-oxidation in the mitochondria and peroxisomes of HK-2 cells. This action also inhibits the HIF-1α-HK2 pathway and glycolysis, ultimately reducing lipid deposition and transdifferentiation in HK-2 cells [79]. Fenofibrate (FF), as a classical PPARα agonist, alleviates lipid toxicity in DKD by enhancing FAO through activation of the PPARα-AMPK-pACC pathway in HK-2 cells [13, 80–82]. Boeravinone C exerts renoprotective effects by activating PPARα, thereby upregulating the expression of carnitine palmitoyltransferase 1 A and acyl-CoA oxidase 1 to regulate lipid metabolism, while enhancing the interaction between p65 and PPARα in the cytoplasm to mitigate inflammatory responses, ultimately attenuating tubular injury in DKD [83]. Guo et al. (2024) [84] further confirmed via in vivo and in vitro models that in db/db mice, dasatinib and quercetin (DQ) reduced the expression of multiple FAO pathway-related proteins while upregulating PPARα. PPARα overexpression subsequently upregulated its downstream FAO pathway target proteins, resulting in increased ATP production, reduced fibrosis in HK-2 cells, and inhibition of renal fibrosis both in vivo and in vitro. Thus, DQ exerts renoprotection in DKD by binding PPARα and enhancing mitochondrial FAO [84]. PPARα activation also regulates cell death. Lin et al. (2022) [85] demonstrated that FGF1^ΔHBS^ treatment directly protects proximal tubular cells in db/db mice from palmitate-induced apoptosis, but this effect is abolished by PPARα inhibition. PPARα serves as a critical downstream effector of FGF1^ΔHBS^-mediated protection against renal tubular cell apoptosis in DKD. Its activation prevents late-stage T2D-induced renal tubular cell death, presenting a promising therapeutic strategy for managing progressive DKD in elderly diabetic patients [85].

Beyond FAO, PPARα influences cholesterol homeostasis. Anthocyanins exert renoprotective effects in DKD by activating the PPARα-LXRα-ABCA1 axis, promoting cholesterol efflux in HG-stimulated HK-2 cells. This also inhibits NF-κB activation and downregulates the expression of proinflammatory cytokines, including TGF-β1, MCP-1, and intercellular adhesion molecule-1 (ICAM-1) [86]. PPARα also protects the kidney by regulating organelle interactions to mitigate the progression of DKD. Diabetic DsbA-L-/- mice exhibited more severe renal tubular injury compared with diabetic mice, accompanied by disruption of mitochondrial-associated endoplasmic reticulum membrane (MAM) integrity and decreased mitophagy [87]. Yang et al. (2023) [87] further demonstrated that helicase with zinc finger 2 (HELZ2), a co-transcription factor that synergizes with PPARα, showed reduced interaction with PPARα in the DsbA-L-deficient conditions. In vitro experiments further confirmed that under HG conditions, overexpression of DsbA-L partially restored the interaction between HELZ2 and PPARα, promoted the expression of mitofusin 2, maintained the integrity of MAM, and enhanced mitophagy in HK-2 cells, thereby alleviating tubular injury in DKD [87]. In type 2 diabetic rats, PPARα activation alleviated renal oxidative stress and reduced the activities of MMP-2 and MMP-9, at least partly by correcting hyperglycemia. These effects prevented the decrease of claudins and demonstrated promising nephroprotective actions in the early stages of DKD [88]. Additionally, PPARα maintains lipid metabolic homeostasis by regulating the integrity of lipid droplet (LD)-mitochondria connections. Yang et al. (2025) [89] observed impaired mitochondrial β-oxidation, severe LD-mitochondria connection disruption, and ectopic renal lipid deposition in HG-treated HK-2 cells, diabetic male mice, and DKD patient renal biopsies. The same study further showed that sesamol maintains the integrity of LD-mitochondria connections by activating the PPARα/PLIN5 pathway, enhancing fatty acid β-oxidation and alleviating lipid deposition in renal tubular cells of DKD [89].

In renal tubular cells, PPARs function at the core intersection of metabolic, inflammatory, and fibrotic pathways. PPARα serves as the primary metabolic regulator mitigating lipotoxicity, while PPARγ exerts potent anti-fibrotic and anti-inflammatory effects. Metabolic dysfunction in tubular cells represents the upstream trigger initiating the vicious cycle of inflammation and fibrosis. Tubular injury and inflammation not only induce interstitial fibrosis but also cause microvascular dysfunction and capillary rarefaction via the secretion of multiple factors. This vascular abnormality is not only a consequence of the initial metabolic injury but also further exacerbates tissue hypoxia and damage, thereby directing our attention to the key regulator of renal microcirculation—the endothelial cell.

### Endothelial cells

In DKD, the body often initiates compensatory mechanisms to counteract injury. Among these, endothelial dysfunction represents a central pathogenic factor in DKD [90]. Adiponectin elevation is an important physiological response. It not only mitigates renal tubular injury in established DKD but also effectively slows DKD progression through its anti-atherogenic and anti-inflammatory effects [91]. The renoprotective mechanisms of adiponectin involve two receptor-mediated pathways: AdipoR1 activates AMPK, while AdipoR2 activates PPARα signaling. This combined action reduces oxidative stress, ameliorates endothelial dysfunction, and upregulates the expression of endothelial nitric oxide synthase [92]. However, persistent endothelial dysfunction can override these protective mechanisms, mainly by aberrantly activating the hypoxia-inducible factor-1α (HIF-1α)/Notch1 pathway. This activation promotes the recruitment of M1 macrophages, contributing to renal injury in type 2 diabetic mice. The PPARα agonist FF targets this imbalance. It improves renal endothelial function in type 2 diabetic mice, directly inhibits the HIF-1α/Notch1 pathway, thereby reducing M1 macrophage recruitment and ultimately protecting against DKD [93]. Furthermore, PPAR agonists, including FF, tesaglitazar, and TZDs, can ameliorate DKD by increasing plasma adiponectin levels and upregulating AdipoR1 and AdipoR2 expression [94–98]. Clinical studies, such as *The Fenofibrate Intervention and Event Lowering in Diabetes* study, confirm that treatment of patients with T2D using FF not only slows DKD progression but also reduces the risk of diabetic retinopathy progression and nonfatal coronary events [13, 99–104].

Endothelial cell dysfunction serves as a central link connecting systemic and renal metabolic disorders to microvascular injury. The activation of PPARα exerts multifaceted protective effects by improving the metabolic milieu, suppressing inflammation (e.g., M1 macrophage recruitment), and directly restoring vascular function. The pathologies of the four key cell types in DKD—podocytes, mesangial cells, renal tubular cells, and endothelial cells—are interconnected via a metabolism-inflammation-fibrosis axis. Members of the PPARs family play critical regulatory roles across these cell types and processes. Therefore, they represent a highly promising therapeutic target for disrupting this cycle and achieving multi-targeted intervention.

## PPARs signaling in DKD: orchestrating metabolic, inflammatory, and fibrotic cascades

### PPARs-mediated metabolic reprogramming and lipotoxicity in DKD

Lipid accumulation-induced lipotoxicity in the kidney represents a significant risk factor for the development and progression of DKD [105]. Various lipid components, such as free fatty acids (FFA), low-density lipoprotein, and ceramides, exert potential nephrotoxicity by targeting podocytes, PTECs, and tubulointerstitial tissues. These lipids exacerbate DKD pathology by increasing ROS generation and lipid peroxidation, resulting in mitochondrial injury and tissue inflammation [106].

In the core metabolic dysregulation of DKD, PPARs, particularly PPARα, play a crucial role by regulating glucose and lipid metabolism [107]. Clinical studies have shown that expression of PPARα and PPARδ is significantly reduced in renal tissues of DKD patients, correlating with a decline in estimated glomerular filtration rate. This suggests that their reduced expression contributes to DKD progression by promoting intracellular triglyceride (TG) accumulation in renal cells [108]. Supporting this, diabetic *PPARA*-knockout mice exhibit severe metabolic dysregulation, characterized by elevated TG and circulating FFA concentrations, along with the exacerbation of DKD, including aggravated glomerulosclerosis and increased albuminuria [109]. Pharmacological interventions targeting this critical pathway show promise. Atorvastatin inhibits TEC injury in the T1DM model with concomitant lipid metabolism disorders by suppressing the expression of miR-21 and upregulating the expression of its downstream gene, *PPARA*. It also protects against DKD by regulating lipid metabolism and PPARα, thereby restoring mitochondrial function [110].

Novel discoveries have emerged in traditional Chinese medicine research regarding multi-target regulation strategies for the PPAR signaling pathway. Huangkui Capsule (HKC), a Chinese traditional medicine extracted from Abelmoschus manihot (L.) medic, has demonstrated significant renal protective effects in clinical practice [111]. In the STZ-induced rat model of DKD, HKC exerted its therapeutic effects by synergistically regulating lipid metabolic pathways. It enhances *PPARA* expression and activates its target genes, such as *lipoprotein lipase* (*LPL*) and *adipocyte fatty acid–binding protein*, which may reduce fatty acid exposure in the kidney [111]. Concurrently, HKC promotes *PPARG* expression along with its target genes, including *acyl-CoA oxidase*, *carnitine palmitoyltransferase-1*, *cytochrome P450 4* *A*, and *LPL*, which are involved in FAO, fatty acid hydroxylation, and fatty acid entry in the liver and kidneys of DKD rats [111].

### PPARs as a master regulator of inflammatory cascades in DKD

The renal expression of ICAM-1, NF-κB, and PAI-1 is significantly upregulated in diabetic rats, indicating that renal inflammation may contribute to the pathogenesis and progression of DKD [112]. PPARs, as pivotal transcription factors, intricately interact with multiple inflammation-related signaling pathways. By modulating the production and release of inflammatory mediators, PPARs play a crucial role in the pathogenesis of DKD [113]. In DKD mouse, PPARα was overexpressed in renal TECs using an AAV9-PPARα vector. This reduced CCL2 expression, suppressed monocyte/macrophage recruitment, and inhibited inflammatory responses mediated by IL-1β, TNF-α [114]. Furthermore, PPARα inhibits necroptosis by downregulating the RIP1/RIP3/MLKL pathway, thereby alleviating HG-induced TECs injury and delaying the progression of DKD [114]. The anti-inflammatory effects of PPARα also extend to macrophage polarization, promoting a shift from M1 to M2 phenotype and reducing the M1 proportion [115]. PPARγ also regulates macrophage polarization. Active vitamin D exerts protective effects in the kidneys of DN rats by promoting the transition of pro-inflammatory M1 macrophages into anti-inflammatory M2 macrophages via the VDR-PPARγ pathway [116]. S-allyl cysteine (SAC) and S-propyl cysteine (SPC) studies indicate PPARα/γ regulate core inflammatory pathways. In diabetic mice, reduced renal PPARα/γ expression was restored by high-dose SAC or SPC intake. This restoration suppressed NF-κB activation, reduced inflammatory factor production, and ameliorated DKD [117]. Additionally, PPARδ activation suppresses HG-induced expression of osteopontin and MCP-1 in STZ-induced DKD models. It achieves this by upregulating the anti-inflammatory repressor B-cell lymphoma-6 in macrophages, thereby inhibiting DKD progression [118].

### PPARs signaling in tubulointerstitial fibrosis and EMT

Tubular tubulointerstitial fibrosis (TIF) and EMT are key pathological features of DKD. Tubular EMT serves as a critical early event initiating TIF, directly driving disease progression and leading to progressive renal function decline in diabetic patients [119, 120]. PPARs, particularly PPARγ, are critical regulators of fibrosis. Preclinical and clinical studies demonstrate that PPARγ activation improves insulin resistance, mitigates renal fibrosis, and alleviates diabetic nephrotic syndrome [121–123]. Activation of PPARγ has been shown to directly ameliorate the EMT process, thereby alleviating TIF [124, 125]. For example, in a recent study using human kidney tissues, C57BL mice, and rat PTECs (NRK52E), PPARγ was found to directly bind to the PTEN gene promoter, thereby transcriptionally activating its expression. This upregulation of PTEN subsequently attenuates the EMT and TIF [120]. In the advanced stages of DKD, elevated levels of cyclin-dependent kinase 5 (CDK5) and its activator p35 are associated with exacerbated TIF and progressive deterioration of renal function. CDK5 activates the extracellular signal-regulated kinase 1/2 pathway, leading to increased phosphorylation levels of PPARγ. This phosphorylation causes a functional shift in PPARγ from an antifibrotic to a profibrotic phenotype, characterized by decreased E-cadherin expression and increased expression of collagen IV and vimentin. These findings suggest that CDK5 may serve as a biomarker for TIF severity and renal dysfunction in DKD [119].

The functional polarity shift is governed by deeper regulatory mechanisms, in which the expression level and transcriptional activity of PPARγ are subject to precise bidirectional modulation by microRNAs. In both in vitro experiments and STZ-induced diabetic rat studies, miR-27a activates the TGF-β/Smad3 pathway by repressing PPARγ. The activation of this signaling cascade significantly promotes the expression of key fibrotic mediators, including CTGF, Collagen I, and fibronectin, ultimately driving the progression of TIF in DKD [126, 127]. Elevated plasma miR-27a levels are associated with deteriorated renal function and exacerbated TIF in the renal tissues of both diabetic rats and patients with DKD [126]. In contrast, in miR-29a transgenic mice, miR-29a functions as a negative regulator of CB1R, thereby blocking the expression of profibrotic and proinflammatory mediators and attenuating renal hypertrophy. MiR-29a overexpression significantly restores reduced PPARγ in diabetic mice [128].

In recent years, the protective role of the endogenous gaseous signaling molecule hydrogen sulfide (H₂S) in DKD has become a research focus. In the STZ-induced diabetic model, H₂S ameliorates DKD by inhibiting renal fibrosis from excessive collagen deposition [129]. Reduced endogenous H₂S synthesis and levels of H₂S-producing enzymes are closely associated with the excessive ECM accumulation, whereas exogenous H₂S supplementation has been shown to ameliorate ECM remodeling [130, 131]. In diabetic mice, exogenous H₂S supplementation can attenuate excessive ECM accumulation by modulating PPAR/RAR-mediated RXR signaling. This regulatory mechanism leads to the downregulation of PAI-1 and MMPs, thereby mitigating renal fibrosis [132].

## Association between PPAR gene polymorphisms and DKD

The association between PPAR gene polymorphisms and DKD has garnered significant attention in recent years (Table 1). The underlying mechanisms primarily revolve around the functional regulation of PPARα and PPARγ isoforms, as well as the differences in renal protective effects mediated by genetic variations. First, the renal protective effects of the *PPARA* have been validated in animal models. In type 1 diabetic *PPARA*-deficiency mice, there is exacerbated glomerular damage, impaired kidney function, and accelerated DKD progression, indicating that PPARα exerts a renoprotective effect [109]. However, the role of *PPARG* genetic polymorphisms, particularly the Pro12Ala variant, in DKD shows significant population heterogeneity and dependence on diabetes type.

**Table 1:** PPAR gene polymorphisms and their associations with DKD.

Gene	Polymorphism	Studied population	Diabetes type	Risk or protective effect	Reference
*PPARA*	Gene Knockout Model	Mice (Animal Model)	T1D	Knockout exacerbated renal injury, indicating a protective effect	[109]
*PPARG*	Pro12Ala	Mixed Population	T2D	Ala12 allele associated with reduced risk, protective association	[133–135]
*PPARG*	Pro12Ala	Chinese	T2D	Ala12 allele associated with increased risk, risk association	[138]
*PPARG*	Pro12Ala	Caucasian	T2D	Pro/Pro genotype increased risk; Ala allele showed no association	[139]
*PPARG*	Exon 2, 6 Polymorphisms	Turkish	T2D	No significant association with DKD	[140]
*PPARG*	Pro12Ala	Mixed Population	T1D	No significant association with DKD	[136, 137]
*PPARD*	PPARD-GLP1R Intergenic Variant	Chinese Han	T2D	Associated with DKD susceptibility	[141]

In patients with T2D, the Pro12Ala polymorphism of *PPARG2* has been demonstrated to be associated with a reduced risk of DKD in several studies. Carriers of the Ala12 allele have a significantly reduced risk of albuminuria, a lower albumin excretion rate, and are associated with slower renal function decline as well as a lower incidence of overt proteinuria [133–135]. By contrast, in T1D patients, the Pro12Ala polymorphism is not significantly associated with DKD risk, suggesting that the protective effect of PPARγ is influenced by diabetes type [136, 137]. Furthermore, this association shows significant population heterogeneity even within the T2D population. For example, in contrast to the aforementioned protective effect, the Pro12Ala polymorphism has been reported to be associated with an increased risk of DKD among Chinese patients with T2D [138]. In Caucasian T2D populations, studies have shown that the *PPARG* Pro/Pro genotype may increase the risk of developing DKD, while the Ala/Ala genotype and the Ala allele do not exhibit such an association in this population [139]. Similarly, studies in the Turkish T2D population have found no significant association between *PPARG* exon 2 or exon 6 gene polymorphisms and the development of DKD [140]. Recent research has also extended to PPAR subtypes beyond PPARγ. For example, a *PPARD-GLP1R* variant has been associated with DKD in Chinese Han individuals with T2D [141].

Ongoing investigations into DKD susceptibility genes may enable early identification of individuals at high risk of disease progression, facilitate the development of personalized therapies for affected patients, and help uncover novel therapeutic targets. Notably, individuals with distinct genetic backgrounds frequently demonstrate differential responses to therapeutic agents, and the identification of disease-predisposing genes also provides critical guidance for advancing pharmacogenomics research.

## Concluding remarks and future directions

DKD, a severe microvascular complication of diabetes, poses a significant threat to patients’ health and quality of life. Profound investigation into its pathogenesis is crucial for improving the prognosis of DKD patients. PPARs play a central regulatory role in DKD, influencing multiple renal cell types and pathological processes. At the cellular level, PPARγ agonists preserve the integrity of foot processes in podocytes, thereby protecting the glomerular filtration barrier [142]. In mesangial cells, the antifibrotic effect of PPARγ can synergize with the lipid metabolism-regulating function of PPARα [56, 57, 63]. In the tubulointerstitium, PPARα activation can alleviate tubulointerstitial injury induced by lipotoxicity [76]. At the pathological mechanism level, PPARs orchestrate the progression of DKD by modulating the “metabolic-inflammatory” cascade. Dysregulated PPARα-driven FAO induces mitochondrial ROS surges, activating the inflammasome. Defects in the PPARγ-adiponectin axis disrupt the AMPK/SIRT1 pathway, leading to the establishment of metabolic memory. PPARβ/δ may regulate TG accumulation and remodel lipid metabolism in renal cells, while also exerting direct anti-inflammatory effects by suppressing HG-induced expression of osteopontin and MCP-1 [108, 118]. These direct cellular effects may subsequently influence the ultimate fibrotic process by modulating macrophage polarization. The functional heterogeneity of PPARβ/δ likely stems from the selective activation of distinct signaling pathways across various renal parenchymal cells. However, significant knowledge gaps remain regarding its role in DKD compared to the α and γ subtypes. Its cell-specific functions in various renal cells, such as tubular epithelial cells and podocytes, remain poorly defined, and evidence of functional crosstalk with other PPAR subtypes is still lacking. Therefore, elucidating the cell-specific actions and regulatory mechanisms of PPARβ/δ will not only uncover novel aspects of DKD pathogenesis but also provide a rationale for developing multi-target therapies against the “metabolism-inflammation-fibrosis” axis. Although studies on PPAR gene polymorphisms and DKD susceptibility have provided new perspectives on disease risk prediction, both functional validation and clinical translation remain significant challenges.

PPARs exert diverse functions across key pathological stages and distinct renal cell types in DKD, making them ideal cell-specific therapeutic targets [34, 114, 143, 144]. However, current PPAR-targeted therapies in the field of DKD face significant challenges. First, the safety profile of existing PPAR modulators constitutes a major bottleneck for their clinical application. For example, the classic TZDs, which are PPARγ agonists, illustrate this issue. Despite their well-established insulin-sensitizing effects, TZDs can induce serious adverse reactions, including bodyweight gain, fluid retention, elevated risk of fractures, and congestive heart failure [145–149]. This safety concern is particularly pronounced in the DKD patient population. Second, current PPARs modulators have non-selective tissue distribution, resulting in systemic side effects, such as increased risk of heart failure. Moreover, single-pathway interventions cannot disrupt the self-reinforcing “metabolism-inflammation-fibrosis” vicious cycle. Emerging mechanisms further exacerbate this therapeutic dilemma: for instance, branched-chain amino acid (BCAA) metabolic dysregulation drives renal fibrosis via activating mTOR signaling, and CNPY2 promotes renal tubular injury in DKD by regulating MAM integrity and ferroptosis through activation of the PERK/ATF4/CHAC1 pathway. Although these pathways ultimately converge on the “metabolism-inflammation-fibrosis” axis, they cannot be effectively targeted by single PPAR agonists, thereby highlighting the limitations of current targeted strategies [150, 151]. Therefore, overcoming these limitations requires a synergistic and innovative strategy. First, leveraging nanotechnology to achieve cell-type-specific delivery. For instance, to overcome the drawbacks of free resveratrol, including its poor bioavailability, low water solubility, and toxicity at high concentrations, these resveratrol-gold nanoparticles not only achieve cell-type-specific delivery through nanotechnology, thereby significantly enhancing antioxidant efficacy, but also effectively mitigate HG-induced oxidative stress, thus expanding resveratrol’s biological applications [152]. Mesenchymal stem cell-derived small extracellular vesicles can precisely deliver CK1δ/β-TRCP, thereby promoting YAP ubiquitination and degradation. This disrupts the fibrosis niche driven by the TGF-β_1_^+^Arg1^+^ macrophage subpopulation [153]. Second, adjunct technologies should be leveraged to develop next-generation PPARs modulators. A primary strategy to reduce the side effects and improve efficacy of current PPAR agonists is to achieve a balanced efficacy-tolerability profile [12]. While specific PPAR-γ agonists forcefully target insulin resistance, they carry prohibitive risks of edema and heart failure [145, 149]. Subsequent dual agonists (e.g., PPAR-α/γ modulators) also failed to resolve these safety concerns due to residual PPAR-γ-mediated toxicities [154]. Compared to these selective or dual-receptor modulators, pan-PPAR agonists (such as chiglitazar) represent a safer and more comprehensive therapeutic strategy. By targeting all three isoforms (α, γ, and δ), pan-PPAR agonists orchestrate systemic glucolipid homeostasis, while simultaneously influencing fibrogenesis and inflammation [155–157]. Crucially, a multi-target combination strategy is indispensable. This encompasses both the combination of different drugs (e.g., co-administering a BCAA homeostasis modulator with a PPARα agonist) and the multi-target synergistic effects of a single agent. For instance, the natural compound curcumin is a typical representative of the latter. It concurrently inhibits multiple critical signaling pathways, including PI3K/AKT/mTOR, TGF-β/Smad, JNK/p38, and NF-κB, demonstrating potent synergistic effects in anti-inflammation, anti-fibrosis, and anti-oxidative stress [158]. Thereby, it exerts multifaceted protective actions against DKD. Although PPAR-targeted therapy is promising for DKD, critical knowledge gaps remain. Future research should focus on the following key directions: 1) elucidating the precise molecular mechanisms of PPAR subtypes in the cellular microenvironment; 2) assessing the long-term biosafety and in vivo targeting efficiency of advanced delivery systems (e.g., nanoparticles); 3) validating the renal specificity and efficacy of next-generation modulators (e.g., pan-PPAR agonists and SPPARMs) in relevant DKD models; 4) accelerating the acquisition of preclinical pharmacodynamics and early clinical data on emerging therapeutic targets (e.g., BCAA metabolism, ferroptosis pathways) to support combination therapies; 5) deepening functional studies on PPARs’ gene polymorphisms for clinical risk stratification and personalized treatment strategies; 6) elucidating the functions of PPARβ/δ in DKD in distinct renal cell types.

In summary, this review distinguishes itself from the existing literature on PPARs in DKD by offering a novel, cell-type-specific integrative perspective. We systematically elucidate the complex regulatory networks of PPARα, PPARγ, and PPARβ/δ in different renal cell types. Furthermore, we propose novel therapeutic strategies that use nanotechnology for cell-specific targeting and multi-target synergistic interventions. For example, concurrently modulating PPARs signaling and ferroptosis pathways may disrupt the vicious cycle of “metabolism-inflammation-fibrosis”. This work lays a theoretical foundation for designing next-generation precision therapies for DKD and charts a path from mechanism to clinical application.

Looking forward, future research should prioritize the precise delineation of regulatory mechanisms for individual PPAR isoforms and their downstream signaling pathways. The integration of advanced technologies, particularly nanoparticle-based drug delivery, together with continued exploration of multi-target strategies, is key to developing more effective precision therapies for DKD.
